# ECOD: integrating classifications of protein domains from experimental and predicted structures

**DOI:** 10.1093/nar/gkae1029

**Published:** 2024-11-20

**Authors:** R Dustin Schaeffer, Kirill E Medvedev, Antonina Andreeva, Sara Rocio Chuguransky, Beatriz Lazaro Pinto, Jing Zhang, Qian Cong, Alex Bateman, Nick V Grishin

**Affiliations:** Department of Biophysics, University of Texas Southwestern Medical Center, 5323 Harry Hines Blvd. Dallas, TX, 75390-8816 USA; Department of Biophysics, University of Texas Southwestern Medical Center, 5323 Harry Hines Blvd. Dallas, TX, 75390-8816 USA; European Molecular Biology Laboratory, European Bioinformatics Institute (EMBL-EBI), Wellcome Genome Campus, Hinxton, Cambridgeshire CB10 1SD, UK; European Molecular Biology Laboratory, European Bioinformatics Institute (EMBL-EBI), Wellcome Genome Campus, Hinxton, Cambridgeshire CB10 1SD, UK; European Molecular Biology Laboratory, European Bioinformatics Institute (EMBL-EBI), Wellcome Genome Campus, Hinxton, Cambridgeshire CB10 1SD, UK; Eugene McDermott Center for Human Growth and Development, University of Texas Southwestern Medical Center, 5323 Harry Hines Blvd. Dallas, TX, 75390-8591, USA; Harold C. Simmons Comprehensive Cancer Center, University of Texas Southwestern Medical Center, 5323 Harry Hines Blvd. Dallas, TX, 75390- USA; Department of Biophysics, University of Texas Southwestern Medical Center, 5323 Harry Hines Blvd. Dallas, TX, 75390-8816 USA; Eugene McDermott Center for Human Growth and Development, University of Texas Southwestern Medical Center, 5323 Harry Hines Blvd. Dallas, TX, 75390-8591, USA; Harold C. Simmons Comprehensive Cancer Center, University of Texas Southwestern Medical Center, 5323 Harry Hines Blvd. Dallas, TX, 75390- USA; European Molecular Biology Laboratory, European Bioinformatics Institute (EMBL-EBI), Wellcome Genome Campus, Hinxton, Cambridgeshire CB10 1SD, UK; Department of Biophysics, University of Texas Southwestern Medical Center, 5323 Harry Hines Blvd. Dallas, TX, 75390-8816 USA; Department of Biochemistry, University of Texas Southwestern Medical Center, 5323 Harry Hines Blvd. Dallas, TX, 75390-9038, USA

## Abstract

The evolutionary classification of protein domains (ECOD) classifies protein domains using a combination of sequence and structural data (http://prodata.swmed.edu/ecod). Here we present the culmination of our previous efforts at classifying domains from predicted structures, principally from the AlphaFold Database (AFDB), by integrating these domains with our existing classification of PDB structures. This combined classification includes both domains from our previous, purely experimental, classification of domains as well as domains from our provisional classification of 48 proteomes in AFDB predicted from model organisms and organisms of concern to global health. ECOD classifies over 1.8 M domains from over 1000 000 proteins collectively deposited in the PDB and AFDB. Additionally, we have changed the F-group classification reference used for ECOD, deprecating our original ECODf library and instead relying on direct collaboration with the Pfam sequence family database to inform our classification. Pfam provides similar coverage of ECOD with family classification while being more accurate and less redundant. By eliminating duplication of effort, we can improve both classifications. Finally, we discuss the initial deployment of DrugDomain, a database of domain-ligand interactions, on ECOD and discuss future plans.

## Graphical Abstract

**Graphical Abstract:**
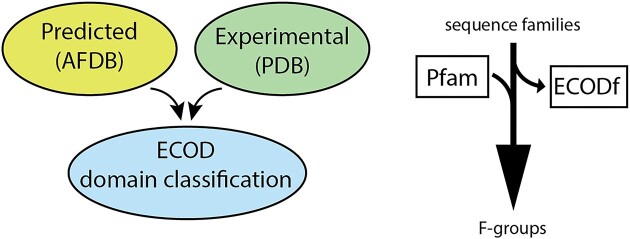

## Introduction

Proteins can be partitioned into domains, units of conserved topology and function. Domain classifications cluster these domains and their homologs into a hierarchical taxonomy. These classifications have been divided into two categories: (i) those principally based on sequence such as Pfam (1), PANTHER (2), SMART (3) and CDD (4) and (ii) those principally based on structure such as SCOP (5), CATH (6) and ECOD (7,8). Where structural data about a protein and its domains exists, more distant homology can sometimes be detected. However, the advent of highly accurate structure prediction methods has eroded this boundary between structure and sequence classifications. These methods, such as AlphaFold (9) and RoseTTAFold (10), have been shown to yield accurate structure predictions at scale (11). Through the AlphaFold Structure Database, >200 M predicted protein structures have been released, including many proteins and protein families that have not previously been structurally characterized (12). Structure classifications, previously designed to accommodate tens to hundreds of thousands of depositions per year, have been prompted to radically adapt to the rapidly changing landscape of available structural data (13). The challenge is to integrate domains from this new set of structural data with previously classified experimental domains in a way that expands existing classifications while guarding against potential errors or limitations in prediction methods.

The evolutionary classification of protein domains (ECOD) is a structural classification that has been actively updated for over a decade (7). Initially forked from SCOP v1.75 (14), ECOD features a hierarchy that emphasizes distant homology over shared topology. ECOD X-groups recognize sets of domains where some weak to moderate evidence exists for homology. Within X-groups, ECOD homologous groups (H-groups) contain those domains with strong evidence of homology. ECOD also explicitly recognizes the potential for homologs to possess distinct topologies; T-groups within an H-group separate homologous domains with topological differences. Finally, family groups (or F-groups) define domains with significant (detectable) sequence similarity that are homologous. These groups are defined automatically (i.e. not curated) by searches against an external library of Hidden Markov models (HMMs). ECOD has solely classified experimental structures up to this point, although we have released numerous pilot classifications of predicted structures. We have classified the human (15), *Vibrio parahaemolyticus* RIMD (16) and *Salmonella enterica typhi* proteomes (17). Having demonstrated the utility of our AlphaFold-specific domain partition and assignment method (DPAM) on these targets, we proceeded to classify 48 whole proteomes released by AFDB (18). We were able to classify 90% of the residues in these proteins into existing ECOD homologous groups.

ECOD sequence families (F-groups) have previously been classified against the ECODf sequence family database (19). ECODf is a collection of HMM models, built from and modified by Pfam and CDD, to separate domains in T-groups into more manageable and understandable groups. They allow us to curate very large groups more easily, and to better understand how small changes in active sites or functional motifs divide closely homologous domains. Because classification into F-groups is ‘downstream’ from expert curation, the generation of F-groups is not affected by curation, it is entirely the output of an automated process. For various reasons, including the difficulty in keeping this database current, and resolving difficult families, we have moved away from using ECODf in this and future ECOD versions. Instead, we now classify F-groups in ECOD against Pfam, one of the initial sources of ECODf domains. By using the most up-to-date version of Pfam, we use one of the most trusted sequence domain classifications to maintain our F-groups. Additionally, through active collaboration, distant homology data from ECOD has been used to both modify Pfam domain boundaries and to curate their ‘Clans’ collection of distant sequence families (see paper describing Pfam in this issue [Paysan Lafosse *et al.* 2024]). Finally, examining the Pfam classification and its associated collection of clans helps to resolve ECOD inconsistencies.

Here we describe our updated ECOD website that incorporates domains from experimentally derived protein structures and computational models sourced from the PDB and AFDB. We illustrate the approaches by which these domains can be identified and either included or excluded from data. From version 290 (20231128) onwards, ECOD F-groups were reclustered, renamed and re-accessioned based on Pfam v37.0. We briefly describe the differences observed during the reclassification and highlight how these differences are reflected on the webpage and in our distributable text files. The incorporation of Pfam led to the definition of several new F-groups (based on existing Pfams) that could only be anchored with a domain from a computed structural model. We discuss these new ECOD families and place them in context with other F-groups.

### Incorporation of domains from predicted structures of proteins in ECOD

ECOD has incorporated domains from both experimental structures in the PDB and predicted structures in the AFDB. We have published a series of provisional ECOD classifications for subsets of proteins based on depositions in AFDB from a set of 48 proteomes spanning model organisms and organisms of importance to global health (18). This provisional classification was made using a purpose-built domain parser for AlphaFold models (DPAM) against an ECOD reference set entirely composed of domains from experimental models (20). ∼90% of the residues in these proteomes could be assigned to existing ECOD homologous groups. Critically, these classifications were built to evaluate domains in structure predictions of proteins and develop our tools and workflows for their classification. These classifications lacked a key feature of our core ECOD classification of PDB structures, in that their domains did not ‘feedback’ or update the core classification. We developed a backend schema and frontend web interface able to incorporate both domains from experimental structures as well as computationally predicted ones. When domains from predicted structures are classified side-by-side with those from experiment, we can (i) designate domains from predicted structures as manual representatives (which can anchor new groups in our classification) and (ii) use those domains to define new hierarchical groups (usually sequence families or F-groups) that have not yet been observed in experimental structures. Ultimately, in this work we did not define new H/T-groups or representatives from predicted structures, and instead focused on developing F-groups and their representatives.

The primary ECOD classification now consists of protein domains from structures both determined by experiment (deposited in the PDB) and the 48 proteomes with structures predicted by AlphaFold2 (deposited in the AFDB). In total, 1.83 M domains from over 1 M proteins compose this combined ECOD classification (Table 1).

**Table 1.** Proteins, domains and families from recent ECOD versions

Version	Domains	Proteins	Families	Family source	Source
v287 (2022-10-14)	966 100	642 096	15 353	ECODf	PDB
v288 (2023-03-09)	1 007 638	670 273	15 359	ECODf	PDB
V289 (2023-05-22)	1027 105	683 225	15 383	ECODf	PDB
v290 (2023-11-28)	1 083 071	718 440	15 406	ECODf	PDB
v291 (2024-03-25)	1 083 021^a^	718 437	11 634	Pfam 36.0	PDB
v292 (2024-08-30)	1 816 770	1 042 189	16 299	Pfam 37.0	PDB/AFDB^b^

^a^No additional PDB structures were added in the creation of v291.

^b^v292 included domains from our previous AFDB 48 proteomes classifications.

**Figure 1.**
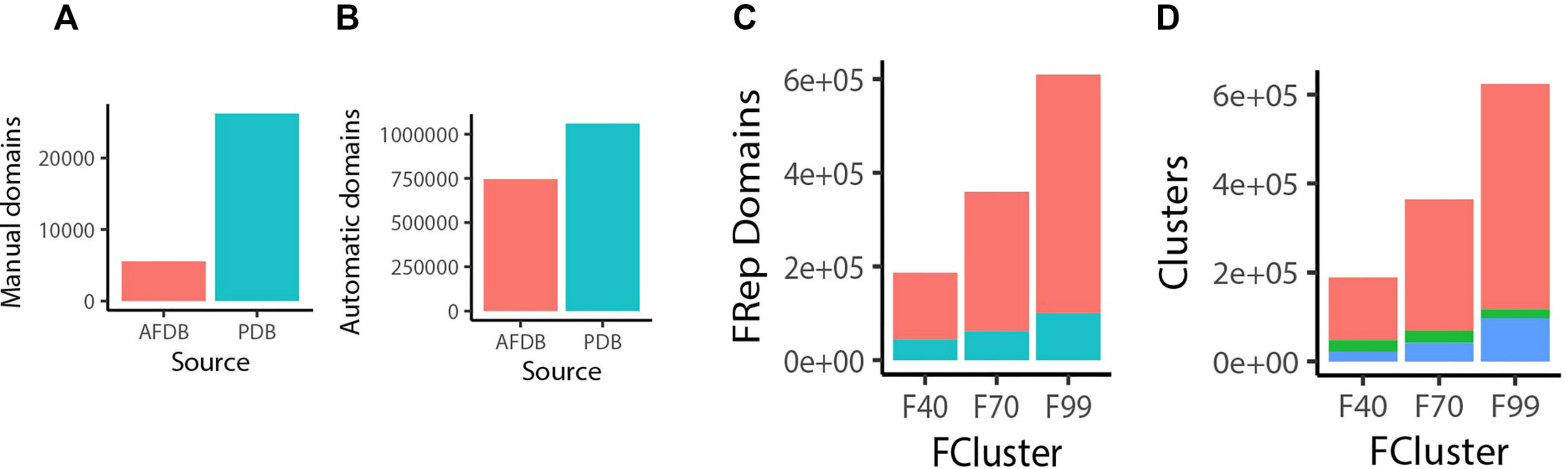
Contribution of AFDB domains to ECOD and its representative clusters. (**A**) Distribution of curated domains (manual representatives) in AFDB 48 proteomes and ECOD. (**B**) Distribution of automated non-representative domains in ECOD by AFDB and PDB. (**C**) Domains source of ECOD cluster representatives for F40, F70 and F99 levels. (**D**) Cluster composition of FClusters in ECOD. Sequence clusters tend to be predominantly composed solely of predicted structure or experimental models, with a comparatively lower fraction of mixed clusters.

Of the 31 750 expert-curated domains that anchor the set, 83% arise from experimental structures (Figure 1A**)**. X, H and T groups remain principally defined by domains from experimental structures. The 733 875 domains from AFDB predicted structures now comprise 41% of ECOD’s total domain content (Figure 1B). Domains from predicted structures were most commonly observed to modify the ECOD hierarchy among the sequence families (F-groups). Of 16 300 F-groups, 4651 are represented by AFDB domains. As previously noted, we provide clustered representative sets derived from ECOD F-groups at 40%, 70% and 99% sequence redundancy levels (7). Representatives of these ‘FClusters’ are selected with a preference for experimental structures where possible and higher average pLDDT among AFDB domains when no experimental structures are available. At every level of clustering there are now more AFDB than PDB cluster representatives (Figure 1C). Additionally, these clusters tend to be principally composed entirely of PDB or AFDB domains, with only a small fraction of clusters containing domains from both experimental and predicted structures (Figure 1D). We anticipate further creation and modification of the ECOD hierarchy as we incorporate additional AFDB domains. The original ECOD PDB classification, as well as the various provisional AFDB classifications, will remain available on the ECOD website. The ECOD AFDB/PDB site is now the default classification for new visitors. The legacy ECOD PDB website (and its distributable files) can be accessed at a new location (http://prodata.swmed.edu/ecod/index_pdb.php).

### Integration of Pfam sequence families into ECOD

ECOD classifies domains on multiple hierarchical levels, although the broader levels such as the X and H-groups are curated using a combination of manual and automated methods, our F-group level is automatically assigned against a library of sequence family profiles. Sequence families can imply shared functions, but that is not necessarily the case. In its early versions, ECOD generated F-groups by comparison to Pfam: following classification to T-groups, domains were partitioned into F-groups by JackHMMER searches. We require that each F-group contains at least one curated representative. Where all domains in newly created F-groups were assigned by homology to domains in other sequence families, one of those new domains was chosen as a provisional curated representative for that group. These F-groups were the basis for the creation of our clustered domain sets, which require that at least one domain from each F-group is present. Over time, we found that some new ECOD domains could not be classified against Pfam, or that differences between domain boundaries in Pfam and ECOD led to anomalous results (e.g. domains from multiple H-groups assigned to the same multi-domain Pfam). For a time, we maintained our fork of Pfam (ECODf), which allowed us to add new sequences and split existing multi-domain sequence models (19). Ultimately, this duplication of effort was unproductive, and we chose instead to enter direct collaboration with Pfam. For some time, ECOD domain boundaries have informed the generation and maintenance of Pfam families. Now, we have deprecated the ECODf sequence family database and have migrated ECOD F-groups to direct generation from Pfam sequence families.

**Figure 2.**
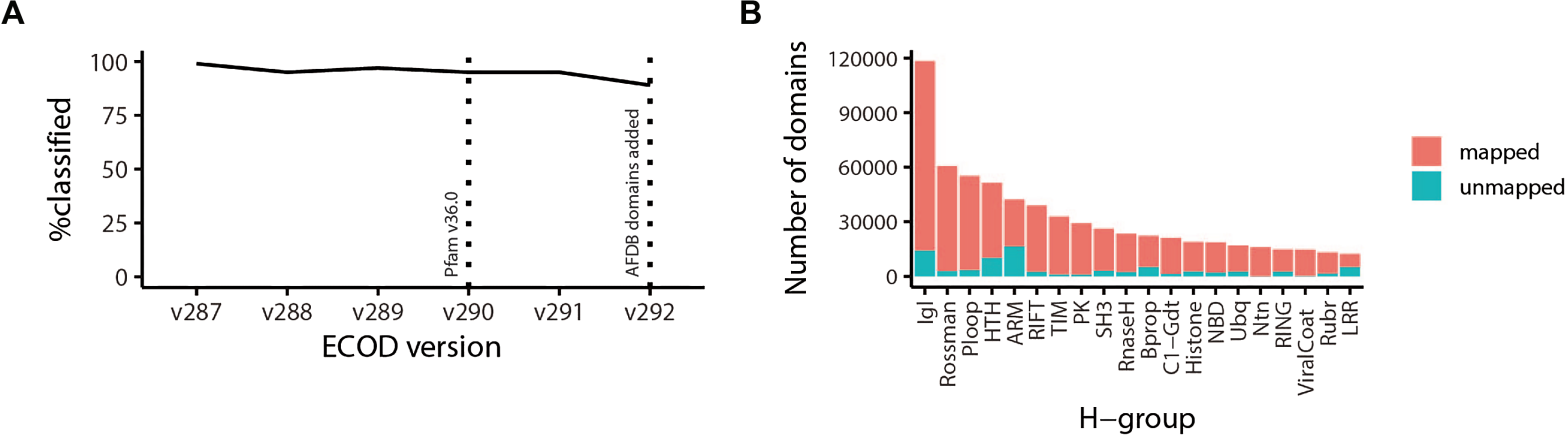
Effect of adding Pfam classification and AFDB domains to ECOD classification. (**A**) Overall percent of ECOD domains classified into sequence family groups (F-groups) in recent ECOD versions. In version 290, ECOD switched from our previous HMM library, ECODf, to directly classifying using Pfam. (**B**) Top 20 most populated homologous groups in ECOD v292 and the relative number of domains mapped to Pfam (magenta) compared to those lacking an F-group classification (cyan).

ECOD v291 was generated against Pfam 36.0 using only domains from PDB structures (Figure 2A). Subsequently, Pfam 37.0 was released and was used to classify our combined AFDB/PDB domain set. The initial switch from ECODf to Pfam 36.0 led to a 25% decrease in F-groups (Table 1), principally reflecting the loss of redundant HMMs and a broadening of sequence families. In either case, both ECODf and Pfam classify a similar overall fraction of ECOD, between 90% and 96% of total domains, although the subset of unclassified domains varies, with Pfam tending to classify more domains from smaller H-groups, whereas ECODf tended to classify more domains from highly populated H-groups (such as Ig domains). Overall, a high fraction of ECOD domains are classified into sequence families (F-groups) in the most populated homologous groups (Figure 2B). The overall classification level of domains into F-groups did drop slightly over the switch to Pfam and AFDB classification. In addition to maintaining the preexisting ECOD website methods for identifying these domains that cannot be classified into F-groups, we have made a list of them available on the ECOD AFDB/PDB website along with other types of marginal or difficult-to-classify domains. Pfam will continue to use this list to improve its coverage of ECOD domains. Of the 21 979 sequence families in Pfam v37.0, 10 878 were used at least once in ECOD v291 (PDB). Considering the combined AFDB/PDB ECOD, 14 219 Pfam sequence families are observed in whole or in part at least once. Many appear in multiple ECOD H-groups or composite F-groups (i.e. multiple Pfams in one domain), indicating that there is still divergence between ECOD and Pfam domain definitions. Although we expect increasing levels of harmonization between ECOD and Pfam domains from this increase in structural data, there may still be families or domains (especially with repeats) where domain boundaries differ between classifications for homologous proteins.

### Creation of ECOD groups using the most recent Pfam and AFDB predictions

The collaboration between Pfam and ECOD has led to the creation of numerous Pfam sequence families and ECOD F-groups. In Pfam v37, 2291 families record ECOD hierarchical groups or ECODf sequence families as the source. At the time of writing, over 2942 families, including un-released families, have been generated in Pfam from ECOD seeds. Within ECOD, 2395 F-groups are generated from these ECOD-sourced Pfam families. In addition, 3632 F-groups have an AFDB manual representative that was generated due to the lack of an experimental representative. These F-groups are linked to Pfam families whose experimental structure is either new (and has not yet been incorporated into the PDB side of ECOD) or has yet to be determined. Overall, tighter integration of ECOD with Pfam, as well as the addition of structural predictions, has allowed us to more efficiently classify those domains lacking previous structure determination, and the inclusion of even a limited set of AFDB structures has greatly enhanced the sequence diversity of our ECOD F-groups. Here we present two examples showing how this integration has led to the propagation of domain classifications in both small singleton (i.e. with no obvious homology) domains and in domains in large, diverse superfamilies.

**Figure 3.**
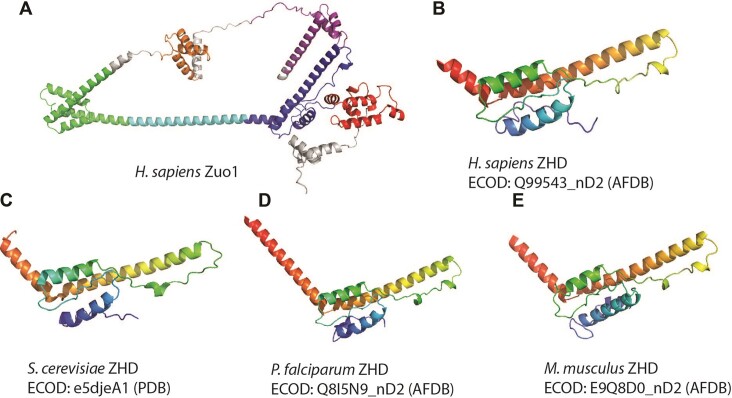
Structures of Zuotin1 Homology domain (ZHD) from experimental and predicted structures. (**A**) The AFDB predicted structure of human Zuo1 (UniProtKB: Q99543) consists of chaperone J-domain (red), ZHD (blue), CHMP-3 linker domain (cyan), a ‘C-terminal Pdr1-activating domain of Zuo1’ (green) and two helix-turn-helix domains (orange and purple). Subsequent to ECOD/Pfam definition of ZUO1-like_ZHD (PF21884), numerous other structurally similar examples of ZHD domain were found in AFDB predicted proteins (**B**–**E**).

Zuotin homology domain (ZHD) (ECOD:1108.1.1.1) is a small helix-strand-helix bundle found within Zuotin, a protein involved in chaperone and post-translation quality control functions in the ribosome-associated complex and a member of a broad class of Hsp40-associated chaperone proteins known as the ‘J-proteins’ (21–23). Zuotin is made up of multiple domains, including a C-terminal 4-helix bundle, a long linking helix, and the (previously uncharacterized) ZHD responsible for mediating contacts with the 60S ribosomal subunit (Figure 3A). ZHD (Figure 3A) was first structurally characterized experimentally (PDB: 5DJE) by X-ray crystallography (23) and subsequently in multiple cryoEM experiments as a component of larger structures (24,25). The initial ECOD domain (ECOD: e5djeA1) was unclassified by Pfam families or the component of a larger Pfam domain and was subsequently used to generate a new sequence family (Pfam: PF21884). When combined with AFDB and Pfam v37, we were able to identify instances of this domain from 31 of the 48 other organisms, such as mouse (Figure 3C), *S. cerevisiae* (Figure 3D) and *P. falciparum* (Figure 3E). This type of domain classification may serve as a foundation in the future for modeling complexes of homologous proteins from different organisms and identifying differences in protein–protein interactions.

**Figure 4.**
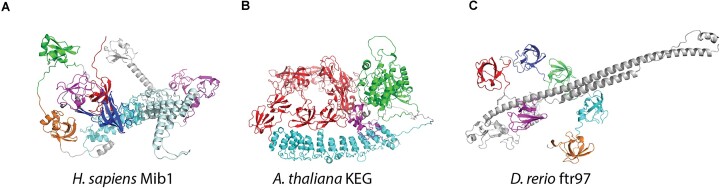
A new family of SH3 domain repeats in E3 ubiquitin ligases are defined through combined efforts between ECOD and Pfam. (**A**) Human Mib1 protein contains four SH3-like repeat domains, two of which are defined as Mib-Herc2 (PF06701) and two which were classified as a new SH3 domain family (SH3_15, PF18346). (**B**) *A. thaliana* KEG E3 ubiquitin ligase, containing a region with seven SH3_15 repeats (red), ankyrin repeat domain (cyan), protein kinase domain (green) and a RING Znf domain (magenta). (**C**) FinTRIM 97 (ftr97), a previously uncharacterized zebrafish protein contains six SH3_15 domains (colored regions).

The SH3-like repeat domains of human Mind bomb (Mib1) protein are substrate recognition domains in this E3 RING ubiquitin ligase (UniProtKB: Q86YT6), involved in ubiquitination of Notch ligands and the subsequent Notch receptor activation (26). These domains were first classified from a series of experimental structures of the MZM-REP region, incorporating two Mib-HERC2 (Figure 4A) domains bordering a RING domain and two (previously unclassified) SH3-like domains in the REP region (27). Although structurally similar to previously classified (Herc2-Mib) SH3 domains (PF06701), the REP SH3 domains were sufficiently distinct by sequence to require a new Pfam family (SH3_15, PF18346), which was subsequently incorporated as an ECOD F-group in the SH3-like domains homologous group (ECOD F: 4.1). This newly defined SH3 domain was subsequently found in many eukaryotic proteomes, including *A. thaliana* KEG E3 ubiquitin ligase (Figure 4B) (28) and an uncharacterized zebrafish protein ftr97 (UniProtKB: Q5RIK1) (Figure 4C) containing multiple SH3_15 repeats and a characteristic E3 ubiquitin ligase pair of RING domains (although no ankyrin repeat). In the classification of the AFDB 48 proteomes an additional 102 instances of this domain were found in predicted structures, especially concentrated in plants (soybean: 39 domains, maize: 24 and *A. thaliana*: 12). This domain classification highlights how combining Pfam and ECOD can help to untangle complicated sequence relationships within large and diverse domain groupings, as well as how the positive feedback from ECOD identifying domains without families and Pfam developing sequence classifications from those domains can fortify both classifications.

### Incorporation of interactions between drugs/small molecules and domains

Recently we launched the DrugDomain database (29) that reports ECOD domains of proteins that are targets for the small molecules and drugs from DrugBank (30). The DrugDomain database not only includes experimentally defined protein structures from the Protein Data Bank but also incorporates AlphaFold models in cases where such structures are unavailable. To enrich AF models with small organic molecules we applied AlphaFIll algorithm (31) that uses protein sequence and structure similarity to retrieve small molecules and ions from experimentally determined PDB structures. Using AlphaFill models, we identify residues with atoms located within 5Å of the DrugBank molecule’s atoms of interest (if present) and map these residues to ECOD domains identified for the entire human proteome via AlphaFold models (18). For each corresponding domain in the ECOD database we specified links to DrugDomain data web pages that include all information about interacting drug/small molecule, protein and ECOD domain annotation.

**Figure 5.**
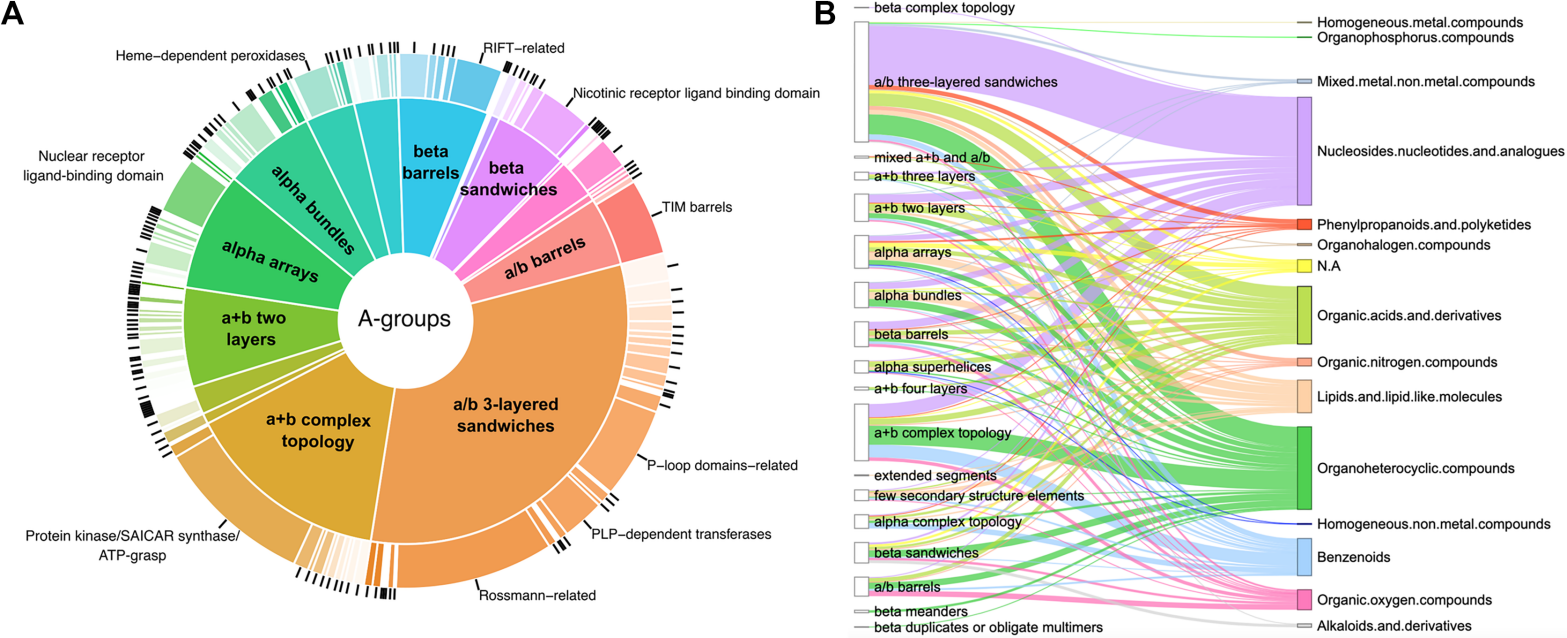
ECOD statistics for AlphaFold models with exact AlphaFill small molecules. (**A**) Distribution of DrugBank molecules interacting with ECOD domains of target AF models. The inside pie shows ECOD architecture groups (A-groups), outside doughnut shows ECOD homology groups (H-groups). (**B**) ECOD A-groups (left column) and superclasses of organic molecules according to ClassyFire classification (36) (right column). Each superclass and lines pointed toward it are denoted by separate color. The thickness of the lines shows the number of ECOD domains interacting with a particular superclass of organic molecules.

Figure 5A shows the distribution of DrugBank molecules interacting with ECOD homologous groups and architectures for AlphaFill models with presented DrugBank molecules of interest. The top three ECOD architectures of the interacting AF domains include α/β three-layered sandwiches, α+β complex topology and α arrays. The α/β three-layered sandwiches architecture is mostly represented by Rossmann-like proteins, which include the Rossmann-related, P-loop domains-related and PLP-dependent transferases H-groups. We previously demonstrated that these proteins bind a majority of the organic molecules superclasses (32,33). Most small molecules interacting with α+β complex topology domains are associated with protein kinases, one of the most druggable protein domains and the domain most frequently encoded among cancer-associated genes (34,35).

The distribution of domains of AF models and superclasses of organic compounds they interact with revealed the top three most common superclasses of ClassyFire (36) classification: Nucleosides, nucleotides and analogues, Organoheterocyclic compounds and organic acids and derivatives (Figure 5B). The largest fraction of domains interacting with compounds from all three superclasses belongs to the α/β three-layered sandwiches ECOD architecture. The majority of this architecture consists of Rossmann-like proteins, which have been shown to bind a wide variety of organic molecule superclasses, with (i) nucleosides, nucleotides and analogs, (ii) organic acids and derivatives and (iii) Organoheterocyclic compounds being the top three (32). For example, Brivudine (DrugBank ID: DB03312, belongs to nucleotides superclass)—is a drug used to treat herpes zoster (37) and Arbaclofen (DrugBank ID: DB08891, organic acids superclass)—is a drug that is used in the treatment of autism (38). The superclass Organoheterocyclic compounds includes such drugs as Atorvastatin (DrugBank ID: DB01076), which is used to lower lipid levels and reduce the risk of cardiovascular disease including myocardial infarction and stroke (39). For the second largest ECOD A-group α+β complex topology (which is mostly represented by kinases) the top three superclasses include: (i) Nucleosides, nucleotides and analogs, (ii) Organoheterocyclic compounds and (iii) benzenoids (Figure 5B). Benzenoids include such drugs as Ibrutinib (DrugBank ID: DB09053), which is an inhibitor of tyrosine-protein kinase BTK, and is used to treat chronic lymphocytic leukemia and mantle cell lymphoma (40).

**Figure 6.**
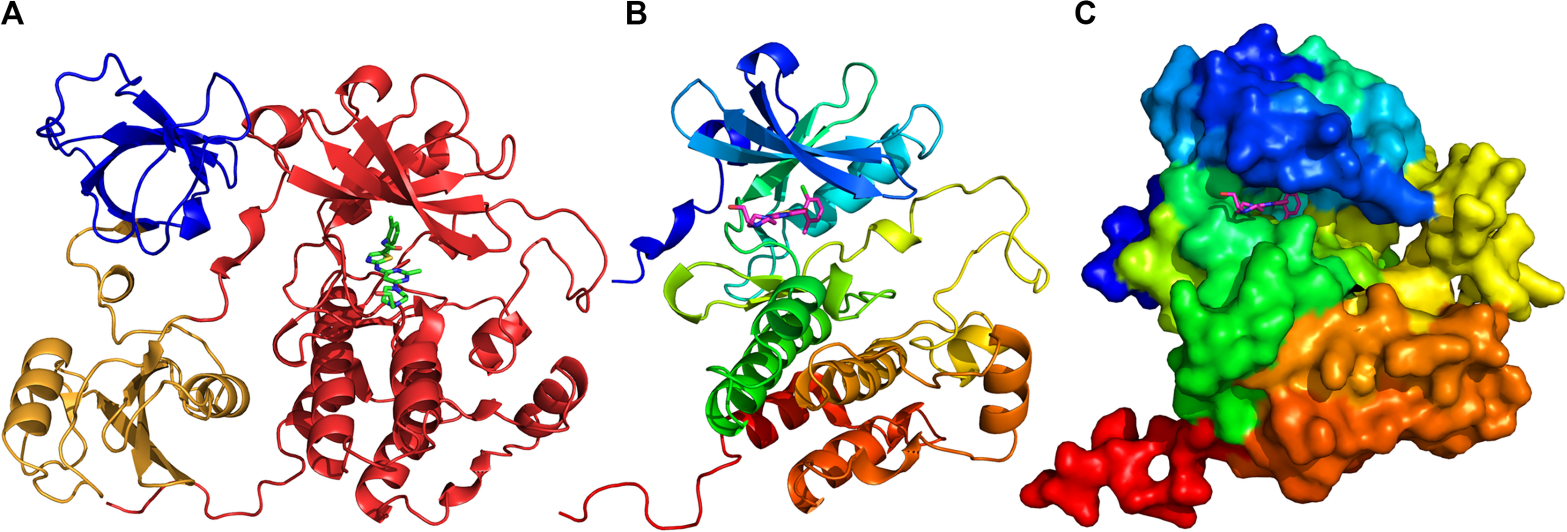
AlphaFold model of tyrosine-protein kinase FRK (UniProt: P42685) with Dasatinib (DrugBank: DB01254). (**A**) Structure of the whole AF model of tyrosine-protein kinase FRK. Following assigned ECOD domains are shown in different colors: SH3 H-group—blue, SH2—yellow, protein kinase/SAICAR synthase/ATP-grasp—red. Dasatinib is colored by elements (C atoms are shown in green). (**B**) Structure of kinase domain of tyrosine-protein kinase FRK AF model colored by rainbow. Dasatinib is colored by elements (C atoms are shown in magenta). (**C**) Surface of the kinase domain of tyrosine-protein kinase FRK AF model colored by rainbow.

Figure 6 shows an example AlphaFold model of tyrosine-protein kinase FRK (UniProtKB: P42685)—the target for Dasatinib (DrugBank: DB01254), filled with the structure of this drug using AlphaFill algorithm. There are no experimentally determined structures for tyrosine-protein kinase FRK, however, its C-terminal domain adopts classical protein kinase fold (Figure 6A). It helped AlphaFill algorithm to position the Dasatinib molecule correctly in the active site of the kinase domain between two subdomains (Figure 6B–C). Dasatinib is a tyrosine kinase inhibitor used to treat acute lymphoblastic leukemia and chronic myeloid leukemia (41,42).

## Conclusions

The widespread release of accurate structure prediction has fundamentally altered the project of domain classification. Previous useful divisions between sequence and structure classification have been eroded as the sequence diversity of structural models has been dramatically increased, and the availability of predicted structures for structures not yet experimentally characterized. ECOD has adapted to this new world by designing a new classification workflow specifically for these predicted models, developing a new database schema no longer specifically designed for PDB structures and collaborating directly with Pfam to develop new classifications for domains with no known family. The future is unclear, the domain classification of experimental protein structures remains necessary, especially as these structures are frequently of protein complexes whose prediction is still beyond the capability of the most modern methods. Certainly, domain classification in the future will not only be the classification of regions of a protein but also the cataloging of those protein–protein interactions that it exhibits, as well as the observed and predicted interactions of those domains with small molecules. Furthermore, the 48 proteomes and their predicted structures that we have integrated with our classification of experimental structures are only a small fraction of existing predicted structures (including the 200 M protein set recently analyzed by Pereira *et al.* [Pereira]). In the immediate future, ECOD will expand to incorporate the remaining curated ∼500 000 SwissProt entries and their predicted structures, as well as cluster representatives from the aforementioned clustering of the 200 M AFDB set, focusing on those proteins with compact regions with little homology to known domains. Finally, we have presented our ECOD integration with DrugDomain here, our pilot classification of domains and their small molecule interactions. We suspect that the structure prediction rate will only continue to grow, and the classification presented herein will serve as the ECOD classification methodology into the next decade.

## Data Availability

All domain data (ranges, sequences and structures) are accessible through the ECOD webpage (http://prodata.swmed.edu).
